# Comparative analysis of tumor biology and prognosis in mucinous and signet-ring cell colon cancers *versus* classical adenocarcinoma

**DOI:** 10.3389/fphys.2023.1199211

**Published:** 2023-07-31

**Authors:** Yang Liu, Wenxin Yin, Xiaoxia Li, Bowen Li, Fang Liu, Pengcheng Kang

**Affiliations:** ^1^ Department of Clinical Laboratory, The 4th Hospital of Harbin Medical University, Harbin, Heilongjiang, China; ^2^ Department of Hepatopancreatobiliary Surgery, Second Affiliated Hospital of Harbin Medical University, Harbin, Heilongjiang, China

**Keywords:** colon cancers, AC, MAC, SRCC, incidence, characteristics, survival

## Abstract

**Background:** Limited information is currently available on the natural history and prognosis of two distinct histological subtypes of adenocarcinoma (AC) in the colon: mucinous adenocarcinoma (MAC) and signet-ring cell carcinoma (SRCC). Therefore, the aim of this study is to examine the clinicopathological characteristics of colon MAC and SRCC, comparing them to classical AC, using a large cohort of cases from the United States.

**Methods:** Patients diagnosed with colon AC, MAC, or SRCC from the SEER database between 2000 and 2018 were included in our study. Incidence trends, patient demographics, tumor characteristics, treatment, and survival were analyzed.

**Results:** In our study, we analyzed a total of 310,813 patients with colon cancers, including 271,382 cases of classical AC, 34,750 cases of MAC, and 4,681 cases of SRCC. Over the study period, we observed a decline in the age-adjusted incidence rates of colon AC, MAC, and SRCC. Notably, the MAC and SRCC cohorts differed significantly from AC in terms of patient characteristics, tumor locations, and treatment patterns. Patients with MAC and SRCC had poorer survival outcomes compared to those with AC. Factors associated with worse survival included older age, male sex, poorly differentiated tumors, advanced stage, and the presence of MAC or SRCC histology. On the other hand, surgical intervention was associated with improved survival.

**Conclusion:** Our study underscores the significance of recognizing the distinct features and outcomes associated with different histological subtypes of colon cancer. Further research is warranted to delve into the underlying biological traits that contribute to these differences and to develop more tailored treatment strategies.

## Introduction

Gastrointestinal cancers pose a significant global health burden, with over 5.1 million new cases and 3.6 million deaths reported in 2020 alone (Bordry et al., 2021; Sung et al., 2021; Davila and Davila, 2022). Among these cancers, colon cancers are among the most common affecting the gastrointestinal tract. While adenocarcinoma (AC) is the predominant subtype, there are two distinct and relatively rare variants known as mucinous adenocarcinoma (MAC) and signet-ring cell carcinoma (SRCC), characterized by mucin secretion (Arai, 2019; Nagtegaal et al., 2020; Ahadi et al., 2021; Washington et al., 2021). The signet-ring cell component that occupies 50% or more of the lesion distinguishes SRCC from MAC. The histological grade of a tumor can significantly impact its biology and survival, leading to the possibility that these two distinct variants represent different diseases with unique clinical features and prognoses. However, due to their rarity, investigating the clinicopathological characteristics and survival outcomes of colon MAC and SRCC has been challenging, and their clinical significance remains uncertain. In the era of precision medicine, management strategies of malignancies are customized to suit patient and tumor characteristics, rather than using a one-size-fits-all approach. While histological classification is readily available, its clinical implications have been subject to conflicting findings in the literature (Overman et al., 2013; Widmann et al., 2016).

Therefore, this study aims to analyze a large population-based cohort from the United States to comprehensively characterize and compare the clinicopathological features and outcomes of colon AC, MAC, and SRCC. By conducting an in-depth analysis of the clinical and pathological characteristics of these cancers, the study aims to offer valuable insights into their etiology, progression, and therapeutic approaches. Ultimately, the findings from this research may contribute to the development of more targeted and effective diagnostic and treatment strategies specific to the distinct characteristics of these colon cancer subtypes.

## Methods

This study analyzed patients diagnosed with colon AC, MAC and SRCC from the Surveillance, Epidemiology and End Results (SEER)-18 program between 2000 and 2018. The inclusion criteria encompassed cases with primary tumors located in the colon (excluding appendix). Patient data regarding clinicopathological characteristics were collected for comprehensive analysis, including variables such as age at diagnosis, gender, race, year of diagnosis, tumor grade, stage, treatment, overall survival (OS), and cancer-specific survival (CSS). Patients with incomplete survival outcome information were excluded from the study. The study received approval from the institutional review board (IRB) of the fourth Hospital of Harbin Medical University, and informed consent was waived due to the observational design.

### Statistical analysis

Incidence rates were calculated using SEER*Stat software and were age-adjusted to the 2000 U.S. standard population and expressed per 100,000 person-years. Categorical variables were presented as number and percentages, and compared by chi-square test. Survival outcomes were estimated using the Kaplan-Meier method with log-rank test. Univariable and multivariable Cox regression analyses were performed to identify potential factors associated with overall survival in colon cancers. Schoenfeld residuals were examined to identify any time-dependent biases. The SPSS and R software was used to perform all tests of statistical significance, with a significance level established at *p* < 0.05. Proportionality of hazards was evaluated for each variable, and schoenfeld residuals were examined to identify any time-dependent biases.

## Results

Between 2000 and 2018, our analysis included 310,813 colon cancer patients who met the inclusion criteria. Of these patients, 271,382 (87.3%) were diagnosed with classical AC, 34,750 (11.2%) were diagnosed with MAC, and 4,681 (1.5%) were diagnosed with SRCC (Table 1).

**TABLE 1:** Comparison of the clinicopathological characteristics of patients with colon AC, MAC, and SRCC.

**Variables**	**Colon cancer**				
	**AC (n=271382)**	**MAC (n=34750)**	**P _(MAC Versus AC)_**	**SRCC (n=4681)**	**P _(SRCC Versus AC)_**
**Gender, n (%)**			0.203		**<0.001**
Male	132966 (49.0)	16900 (48.6)		2507 (53.6)	
Female	138416 (51.0)	17850 (51.4)		2174 (46.4)	
**Age (years), n (%)**			**<0.001**		**<0.001**
<65	111792 (41.2)	13936 (40.1)		2323 (49.6)	
≥65	159590 (58.8)	20814 (59.9)		2358 (50.4)	
**Race, n (%)**			**<0.001**		**<0.001**
White	210553 (77.6)	28258 (81.3)		3805 (81.3)	
Black	35389 (13.0)	4035 (11.6)		462 (9.9)	
Other	25440 (9.4)	2457 (7.1)		414 (8.8)	
**Marital status, n (%)**			0.084		0.628
Married	142311 (52.4)	18052 (51.9)		2438 (52.1)	
Other	129071 (47.6)	16698 (48.1)		2243 (47.9)	
**Year of diagnosis, n (%)**			**<0.001**		**<0.001**
2000-2009	142076 (52.4)	21644 (62.3)		2617 (55.9)	
2010-2019	129306 (47.6)	13106 (37.7)		2064 (44.1)	
**Grade, n (%)**			**<0.001**		**<0.001**
Well differentiated	198435 (73.1)	23512 (67.7)		243 (5.2)	
Poorly differentiated	47016 (17.3)	7136 (20.5)		3502 (74.8)	
Unknown	25931 (9.6)	4102 (11.8)		936 (20.0)	
**Lymph nodes positive, n (%)**			**<0.001**		**<0.001**
Yes	104763 (38.6)	15710 (45.2)		2991 (63.9)	
No	123513 (45.5)	17499 (50.4)		1241 (26.5)	
Unknown	43106 (15.9)	1541 (4.4)		449 (9.6)	
**Stage, n (%)**			**<0.001**		**<0.001**
Localized	106133 (39.2)	9138 (26.3)		523 (11.2)	
Regional	104601 (38.5)	17094 (49.2)		2158 (46.1)	
Distant	60648 (22.3)	8518 (24.5)		2000 (42.7)	
**Surgery, n (%)**			**<0.001**		**<0.001**
Done	243614 (89.8)	31881 (91.7)		3655 (78.1)	
None	27768 (10.2)	2869 (8.3)		1026 (21.9)	

AC: adenocarcinoma; MAC: mucinous adenocarcinoma; SRCC: signet ring cell carcinoma

### Incidence

**FIGURE 1:**
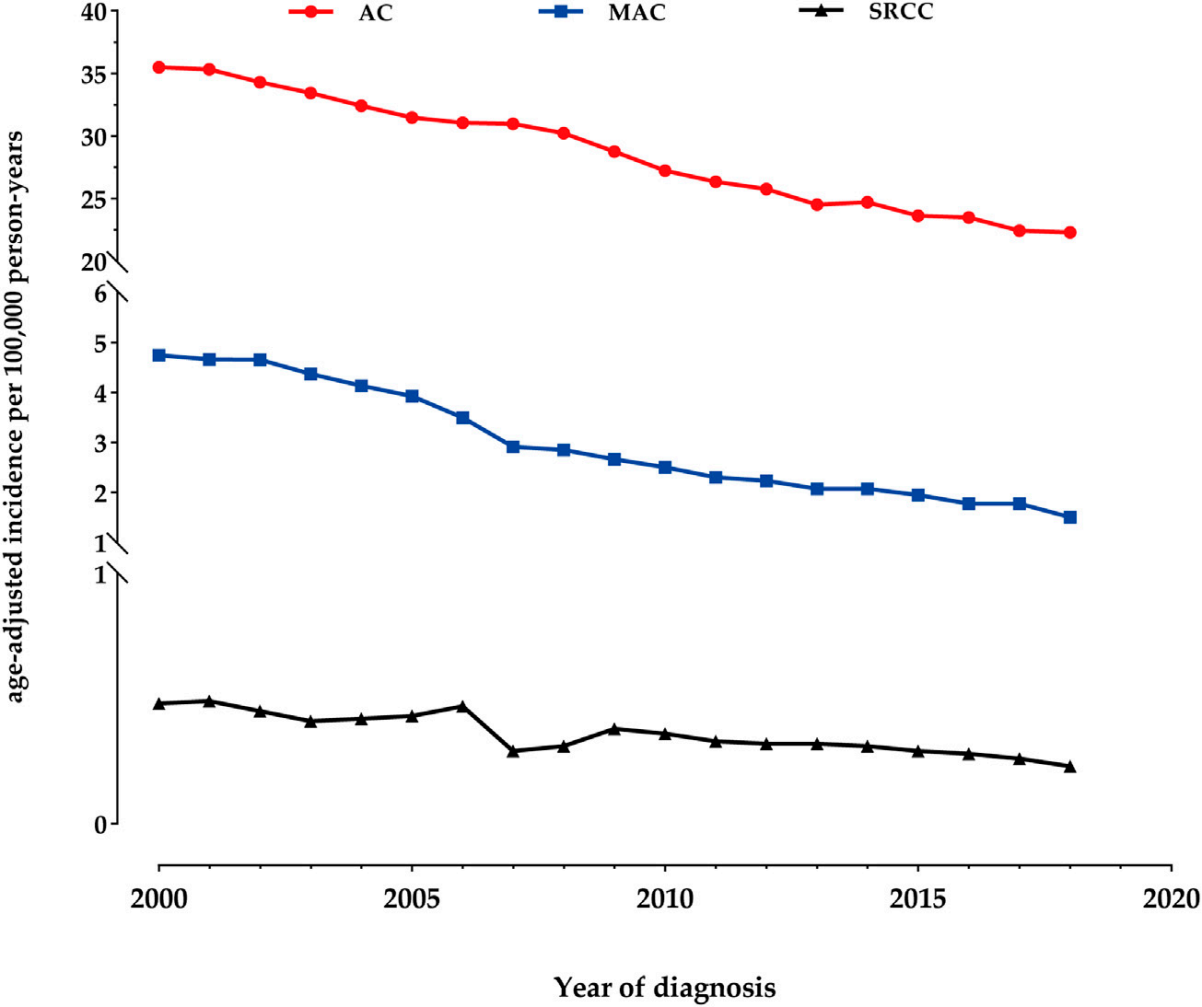
Incidence Trends of colon adenocarcinoma (AC), mucinous adenocarcinoma (MAC), and signet-ring cell carcinoma (SRCC) fom 2000 to 2018.

The overall age-adjusted incidence of AC during the study period was 28.04 per 100,000 person-years. We observed a 1.6-fold decrease in the incidence, from 35.48 per 100,000 person-years in 2000 to 22.30 per 100,000 person-years in 2018. Similarly, the incidence of MAC showed a 3.2-fold decrease, declining from 4.74 per 100,000 person-years in 2000 to 1.50 per 100,000 person-years in 2018. As for SRCC, the age-adjusted incidence was 0.48 per 100,000 person-years in 2000, which decreased to 0.23 per 100,000 person-years by 2018 (Figure 1).

### Patient characteristics

The marital status did not significantly differ among patients with classical AC, MAC, and SRCC. The distribution of sex was similar between AC and MAC patients, while there was a higher proportion of males in the SRCC cohort. Patients with SRCC were more likely to be younger than 65 years old compared to those with AC (49.6% VS. 41.2%, *p* < 0.001). There was a higher percentage of MAC diagnoses between 2000 and 2009 compared to AC diagnoses (62.3% VS. 37.7%, *p* < 0.001). Both MAC and SRCC were more likely to be diagnosed with poorly differentiated tumors and at more advanced stages upon presentation, especially SRCC (*p* < 0.001 for each). Lymph node metastasis was found in 38.6% of AC patients, 45.2% of MAC patients, and 63.9% of SRCC patients. In terms of treatment, patients with SRCC had a significantly lower rate of surgical interventions compared to those with AC (Table 1).

### Stage and histology distribution

**FIGURE 2:**
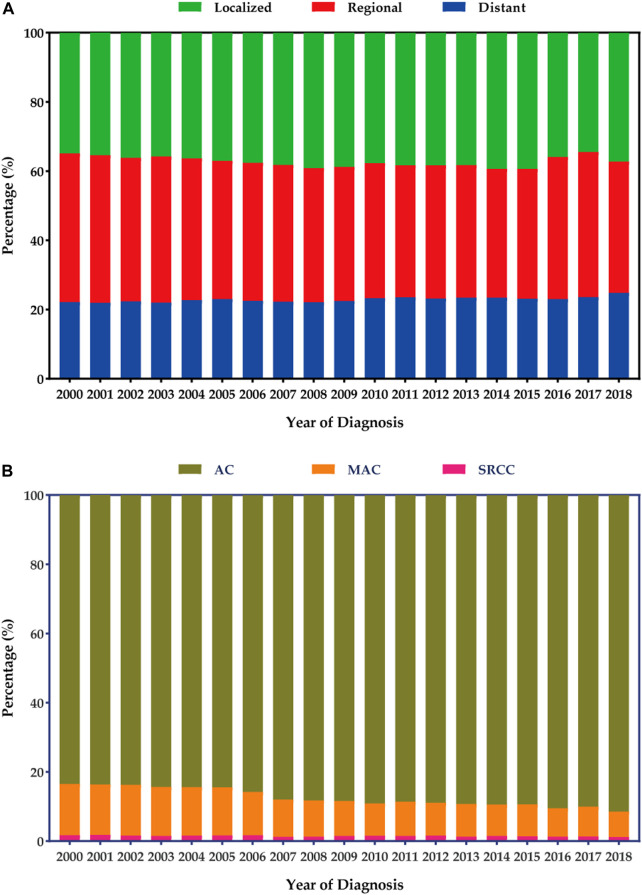
Stage and histology distribution among patients with colon cancers. **(A)** Stage distribution. **(B)** Histology distribution.

Figure 2 presents the stage and histology distribution of colon cancers for each year during the study period. In the overall cohort, the proportions of distant disease showed a slight increase from 2000 to 2018 (Figure 2A). However, in terms of histology distribution, the proportions of classical AC increased from 2000 to 2018, while the proportions of MAC decreased. On the other hand, the proportions of SRCC in the colon remained relatively stable (Figure 2B).

### Survival

**FIGURE 3:**
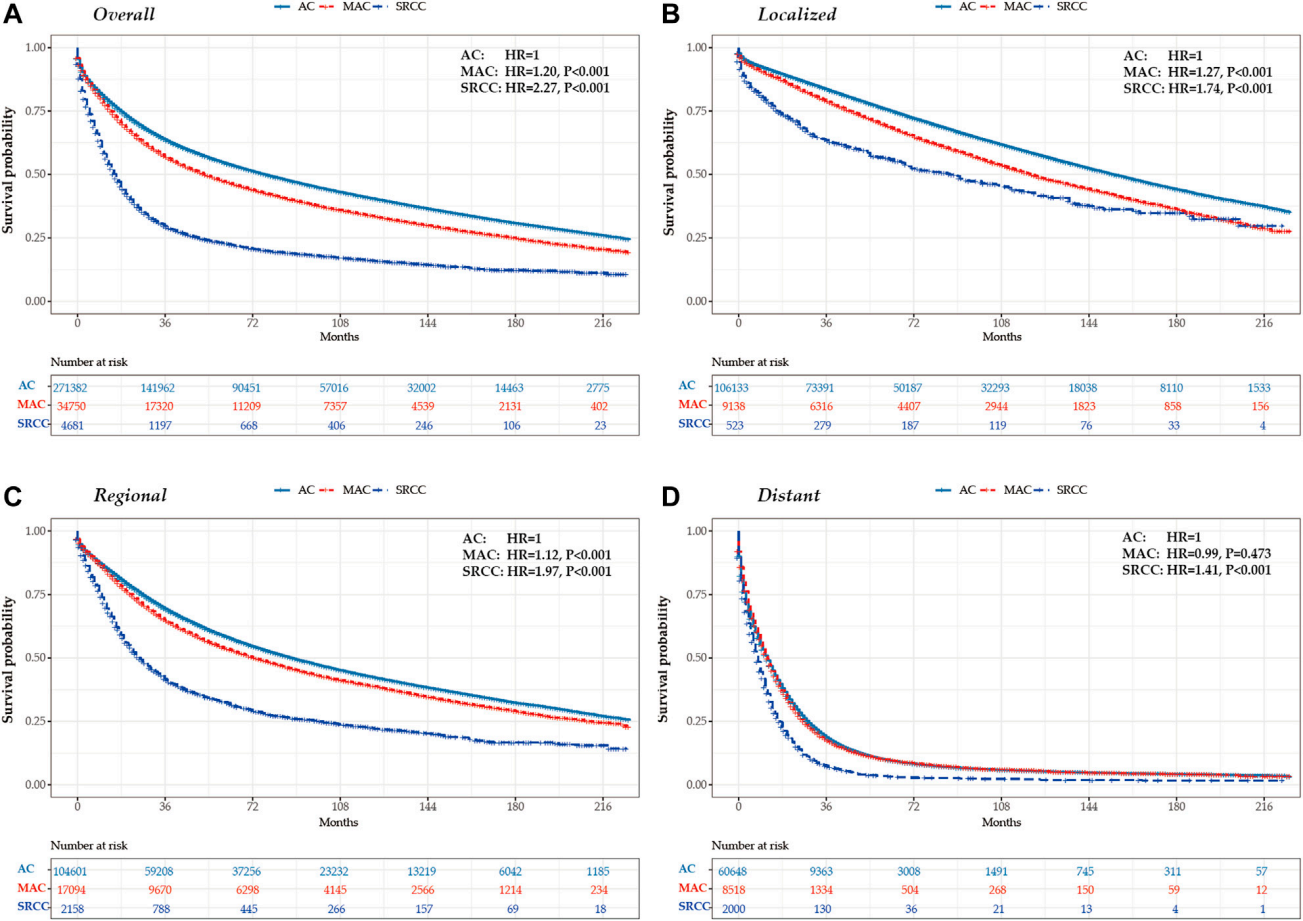
Kaplan-Meier survival curves for colon cancers by histological subtypes and tumor stage in the United States between 2000 and 2018. **(A)** overall **(B)** localized stage **(C)** regional stage **(D)** distant stage.

Table 2; Figure 3 present the survival outcomes for different histological subtypes of colon cancers. For the overall cohort, compared with patients with classical AC (median overall survival, 77.0 months), patients with MAC and SRCC had less favorable survival outcomes, with median overall survivals of 53.0 months and 16.0 months, respectively (HR_MAC_ = 1.20, *p* < 0.001; HR_SRCC_ = 2.27, *p* < 0.001) (Figure 3A). Likewise, patients with loco-regional MAC or SRCC experienced worse survival outcomes when compared to those with loco-regional AC (Figures 3B, C). Among patients with distant disease, those with MAC had similar overall survival to those with AC, both having a median overall survival of 12.0 months (*p* = 0.473). However, the survival outcomes for patients with SRCC were still less favorable, with a median survival of 8.0 months (Figure 3D).

**TABLE 2:** Median, 1-year, 3-year, and 5-year survival rate of Patients with Colon Cancers by histological subtypes.

**Survival**	**AC**	**MAC**	**SRCC**
Median, mo			
Overall	77.0	53.0	16.0
Localized	154.0	121.0	87.0
Regional	88.0	73.0	26.0
Distant	12.0	12.0	8.0
1-year survival, %			
Overall	80.2	77.3	56.1
Localized	91.5	89.7	78.5
Regional	85.2	84.3	69.5
Distant	49.0	49.7	35.8
3-year survival, %			
Overall	63.8	57.2	29.6
Localized	83.6	79.1	63.6
Regional	69.4	65.0	41.7
Distant	18.8	17.9	7.5
5-year survival, %			
Overall	54.7	47.5	23.1
Localized	75.9	69.6	56.8
Regional	58.8	54.1	32.7
Distant	10.0	10.1	3.5

AC: adenocarcinoma; MAC: mucinous adenocarcinoma; SRCC: signet ring cell carcinoma

### Factors associated with OS

Factors impacting overall survival were determined using univariable and multivariable Cox regression analyses. No evidence of violation of the proportional hazard assumption was found. Table 3 presents the clinicopathological variables associated with overall survival in colon cancers. In the multivariable analyses, patients aged ≥65 years were found to have a worse prognosis (HR, 2.39; 95%CI, 2.36–2.41; *p* < 0.001). Female sex was associated with significantly better survival outcomes compared to male sex. It is not surprising that patients with poorly differentiated tumors or more advanced diseases exhibited worse overall survival. Moreover, patients with a histology of MAC or SRCC had less favorable outcomes compared to those with classical AC. Surgical intervention was associated with improved survival outcomes for patients with colon cancers (HR, 0.38; 95%CI, 0.37–0.39; *p* < 0.001).

**TABLE 3:** Prognostic factors in patients with colon cancer in the United States.

**Variables**	**Colon cancer**			
	**Univariable**		**Multivariable**	
	**HR (95% CI)**	**P**	**HR (95% CI)**	**P**
Age				
<65 years	Ref		Ref	
≥65 years	2.01 (1.99, 2.03)	**<0.001**	2.39 (2.36, 2.41)	**<0.001**
Gender				
Female	Ref		Ref	
Male	1.02 (1.01, 1.03)	**0.004**	1.07 (1.06, 1.08)	**<0.001**
Race				
White	Ref		Ref	
Black	1.11 (1.10, 1.13)	**<0.001**	1.15 (1.14, 1.17)	**<0.001**
Other	0.74 (0.72, 0.75)	**<0.001**	0.78 (0.77, 0.80)	**<0.001**
Year of diagnosis				
2000-2009	Ref		Ref	
2010-2018	0.85 (0.84, 0.86)	**<0.001**	0.81 (0.80, 0.82)	**<0.001**
Tumor grade				
Well differentiated	Ref		Ref	
Poorly differentiated	1.65 (1.63, 1.67)	**<0.001**	1.35 (1.33, 1.36)	**<0.001**
Histology				
AC	Ref		Ref	
MAC	1.20 (1.18, 1.21)	**<0.001**	1.09 (1.08, 1.11)	**<0.001**
SRCC	2.27 (2.20, 2.35)	**<0.001**	1.42 (1.38, 1.47)	**<0.001**
Tumor stage				
Localized	Ref		Ref	
Regional	1.62 (1.60, 1.64)	**<0.001**	1.64 (1.62, 1.66)	**<0.001**
Distant	6.63 (6.54, 6.71)	**<0.001**	5.83 (5.75, 5.92)	**<0.001**
Surgery				
No	Ref		Ref	
Yes	0.19 (0.18, 0.20)	**<0.001**	0.38 (0.37, 0.39)	**<0.001**

AC: adenocarcinoma; MAC: mucinous adenocarcinoma, SRCC: signet ring cell carcinoma, HR: hazards ratio, CI: confidence interval, Ref: reference. Bold indicates significance.

## Discussion

To our knowledge, this study represents the largest population-based analysis comparing the epidemiology, clinicopathological characteristics, and outcomes of colon AC, MAC, and SRCC in the United States from 2000 to 2018. The majority of cases in our study were classical AC (87.3%), with MAC and SRCC comprising smaller proportions. During the study period, we observed a 1.6-fold decrease in the overall age-adjusted incidence of AC, from 35.48 per 100,000 person-years in 2000 to 22.30 in 2018. Similarly, the incidence of MAC showed a 3.2-fold decrease, declining from 4.74 per 100,000 person-years in 2000 to 1.50 in 2018. As for SRCC, the age-adjusted incidence was 0.48 per 100,000 person-years in 2000, which decreased to 0.23 by 2018. Furthermore, MAC and SRCC exhibited significantly distinct clinical features compared to classical AC. Both MAC and SRCC were more likely to be diagnosed with poorly differentiated tumors and at more advanced stages upon presentation, particularly SRCC. Patients with SRCC also experienced significantly worse survival outcomes compared to those with AC, even after adjusting for tumor stage.

Gastrointestinal MAC and SRCC are two rare histological subtypes of cancers associated with abundant mucous production (Gopalan et al., 2011; Imai et al., 2013). MAC is presented with a predominant extracellular mucin accumulation, while SRCC, on the other hand, is characterized by excessive intracytoplasmic mucin (Hartman et al., 2013; Barresi and Pedrazzani, 2020). Previous studies have shown significant differences in the clinicopathological features between MAC and SRCC, as well as associations between tumor biology, treatment sensitivities, and survival results (Yang et al., 2004; Nitsche et al., 2013; Nitsche et al., 2016; Kermanshahi et al., 2017; Tang et al., 2020). However, whether these biological traits carry the same weight in cases with colon MAC *versus* SRCC remains unclear and requires further investigation. In addition, the relative paucity of MAC and SRCC cases as compared to typical AC in digestive system has limited characterization of MAC and SRCC to small case series. Our study based on the SEER-18 database met the requirement to ensure the presence of sufficiently large cohorts of study population upon which outcome analyses can be performed.

The impact of MAC and SRCC on different organs of the gastrointestinal tract is unclear, including whether they share similar clinical and pathological features or outcomes. Generally, SRCC tends to be more aggressive, diagnosed at later stages, and have lower survival rates than classical AC (Pernot et al., 2015). Poor tumor differentiation and delayed diagnosis are more common in MAC and SRCC than in conventional AC, which may contribute to these unfavorable characteristics, especially in SRCC (Mekenkamp et al.; Zaafouri et al., 2022). When comparing SRCC to MAC by location, differences in their behavior and prognoses are significant, partially due to the variations in tumor distribution between the two histological subtypes. Consistent with most studies, patients with SRCC have a higher incidence of lymph node metastasis and distant disease (Chew et al., 2010).

The incidence of colon cancer has significantly decreased over the past 2 decades, indicating that the peak of colon cancer may have already been reached. Unhealthy lifestyle habits, including excessive consumption of fat, sugar, red meat, and alcohol, as well as having a high body mass index, are well-known risk factors for gastrointestinal tumors. The observed decline in incidence in our study can be attributed to the promotion of healthier lifestyles and the implementation of effective colon cancer screening programs, enabling the early detection and treatment of precancerous lesions. As a result, the overall incidence of colon cancer has gradually decreased over time.

Although traditional staging systems are effective in predicting survival in colon cancers, tumor histology and pathological grading can provide valuable information about clinical behavior. For instance, a retrospective study by Song et al. demonstrated that the presence of signet-ring cell component in MAC was associated with worse overall and recurrence-free survival (Song et al., 2019). Further research is needed to explore the biological distinctions among these rare subtypes. To the best of our knowledge, our study represents the largest population-level analysis in the United States regarding the clinicopathological characteristics of gastrointestinal MAC and SRCC, confirming previous nationwide epidemiological data from other sources.

Managing MAC and SRCC remains challenging due to their rarity and heterogeneous nature. Treatment typically involves a combination of surgery, chemotherapy, and radiation therapy, tailored based on the tumor location, stage, and the patient’s overall health. However, prior studies have suggested that MAC and SRCC may exhibit an inferior response to commonly used therapies, such as neoadjuvant chemoradiotherapy, when compared to classical AC (Kim et al., 2013; McCawley et al., 2016). Due to its more aggressive behavior and tendency for early metastasis, SRCC often necessitates a more aggressive management approach. Therefore, patients diagnosed with these subtypes may be suitable candidates for personalized treatment, including a multidisciplinary approach and more frequent follow-up. Additional strengths of our study include the extensive longitudinal follow-up spanning nearly 20 years and the analysis of clinicopathological characteristics using individual-level data on a national scale, which allowing us to gain a unique insight into the differences and disparities among colon AC, MAC, and SRCC. Nevertheless, it is important to acknowledge the limitations of our study. Being a retrospective design, we cannot completely eliminate selection biases inherent in the data. Furthermore, the absence of detailed treatment information is another potential limitation that may impact the robustness of our findings.

Our study provides a detailed picture of the clinicopathological features of colon AC, MAC and SRCC based on a large cohort of patients from US. While they share some similarities, they differ significantly in terms of their histological features, clinical behavior, and prognosis. Our findings highlight the importance of distinguishing these subtypes from classical AC and underscore the need for further research to explore the variations in their biological traits. It is important to acknowledge that our study has certain limitations, including potential selection biases and incomplete data on treatment details. Nevertheless, it provides valuable insights into the differences and disparities among colon AC, MAC, and SRCC.

## Data Availability

Publicly available datasets were analyzed in this study. This data can be found here: SEER program.
